# Impact of ivabradine on the cardiac function of chronic heart failure reduced ejection fraction: Meta‐analysis of randomized controlled trials

**DOI:** 10.1002/clc.23581

**Published:** 2021-02-27

**Authors:** Sasmita Bryan Richard, Bi Huang, Gang Liu, Yuan Yang, Suxin Luo

**Affiliations:** ^1^ Department of Cardiology The First Affiliated Hospital of Chongqing Medical University Chongqing China

**Keywords:** chronic heart failure, ejection fraction, ivabradine, LV remodeling, re‐hospitalization

## Abstract

Elevated resting heart rate in chronic heart failure (HF) patients has been associated with higher mortality and poor prognosis. Ivabradine is a new pure bradycardic agent that has been used to treat angina or heart failure reduced ejection fraction (HFrEF) with sinus heart rate above 70 beats per minute. However, the effect of ivabradine for chronic HF patients on rehospitalization and cardiac function is still inconsistent. Thus, this meta‐analysis aimed to elucidate the effect of Ivabradine in chronic HFrEF patients. We systematically searched PubMed, Medline, Clinical Trials.gov, and The Cochrane Central Register of Controlled Trials for randomized controlled trials (RCTs) of ivabradine with search terms Ivabradine (MeSH Terms), chronic heart failure and beta‐blocker. The primary endpoints of the study include the impact of Ivabradine on heart rate, left ventricle ejection fraction (LVEF), left ventricular remodeling, exercise capacity, and quality of life (QoL) in patients with chronic HFrEF. Secondary endpoints were safety analysis of Ivabradine including cardiovascular mortality, worsening HF readmission, visual disturbances, and asymptomatic bradycardia. The analysis was done by Review Manager 5.4 Analyzer, to analyze the mean differences (MD) for continuous data and risks ratio (RR) for dichotomous data. A total of six RCTs and one subgroup analysis showed add of Ivabradine to standard HF therapy was associated with greater resting heart rate reduction (MD = −9.57; 95% CI ‐11.15, −8.00), improved LVEF (MD = 3.89; 95% CI 2.61, 5.17), left ventricular reverse remodeling improvement (MD = −3.73; 95% CI ‐4.25, −3.21, LVESV; MD = −17.00, 95%CI ‐29.65, −4.35, LVEDD; MD = −1.43, 95%CI ‐2.78, −0.08, LVEDV; MD = −14.75, 95%CI ‐34.36, 4.87), increased exercise capacity (exercise duration; MD = 8.52; 95%CI 0.09, 16.94), and significant reduction on rehospitalization due to worsening HF (RR = 0.76, 95%CI 0.69, 0.84). However, Ivabradine has no significant effect on the quality of life (MD = 0.65; 95%CI ‐10.52, 11.82), and cardiovascular mortality (RR = 0.92; 95%CI 0.82, 1.03). Moreover, there were some events of visual disturbances and asymptomatic bradycardia observed in the Ivabradine group compared to the placebo group (RR = 4.76; 95%CI 3.03, 7.48; RR = 3.78; 95%CI 2.77, 5.15, respectively). Addition of Ivabradine to standard HF therapy is associated with cardiac function improvement, reduction on worsening HF readmission, greater HR reduction, and better exercise capacity in chronic HFrEF patients, although it cannot reduce cardiovascular mortality or improve the quality of life.

## 1 BACKGROUND

Elevated heart rate (HR) is an independent risk factor for left ventricular remodeling, poor cardiovascular outcomes, as well as higher all‐cause mortality in patients with cardiovascular disease.^1^, ^2^ Framingham study has shown an increment in all‐cause mortality by 14% at every 10 beats per minute increase in HR, along with 2‐fold increased risk of incident heart failure (HF).^3^ The involved mechanisms are possibly related to increased myocardial oxygen demand, accelerate atherosclerosis event, decrease myocardial blood supply, and shortens diastolic time.^4^, ^5^, ^6^ In patients with HF, previous studies have also show resting HF around 60–70 beats per minute is an important therapeutic goal.^7^, ^8^, ^9^, ^10^

Beta‐blocker is essential for the treatment of HF patients due to their efficiency on cardiac remodeling, reduction of hospital readmission, and cardiovascular death.^11^, ^12^, ^13^ Although beta‐blocker is regarded as the first‐line agent for HF patients, several contraindications, complications, and side effects, such as hypotension, worsening cardiac function, asthma, acute exacerbation of chronic obstructive pulmonary disease may limit its use in clinical practice.^14^, ^15^, ^16^

Ivabradine is the new promising pure bradycardic agent without affecting cardiac conductivity. Ivabradine is selectively acted to lower the HR through specific inhibition of the I_f_ channel in the sinus node, thus resulting in a reduction of HR by prolonging diastolic depolarization of a pacemaker action potential.^17^ Ivabradine was officially approved by US FDA for the treatment of HF in 2015 with indications of heart failure reduced ejection fraction (HFrEF) and sinus rhythm ≥70 beats per minute on a maximal dosage of beta‐blocker or when beta‐blocker is contraindicated.^18^ To date, there are only two large studies conducted the use of Ivabradine in HF patients including BEAUTIFUL study^19^ and SHIFT study^20^; however, both studies had different inclusion criteria and the findings were inconsistent. Several other studies have also reached inconsistent conclusions.^21^, ^22^, ^23^, ^24^ Accordingly, this meta‐analysis was designed to evaluate the safety and efficiency of Ivabradine added to the standard HF treatment in patients with chronic HFrEF.

## 2 METHODS

This study was conducted per standard article publication in Medical Journals, as this article has been made in coherence with Preferred Reporting Items for Meta‐Analysis PRISMA Checklist.^25^

### 2.1 Search strategy

**FIGURE 1:**
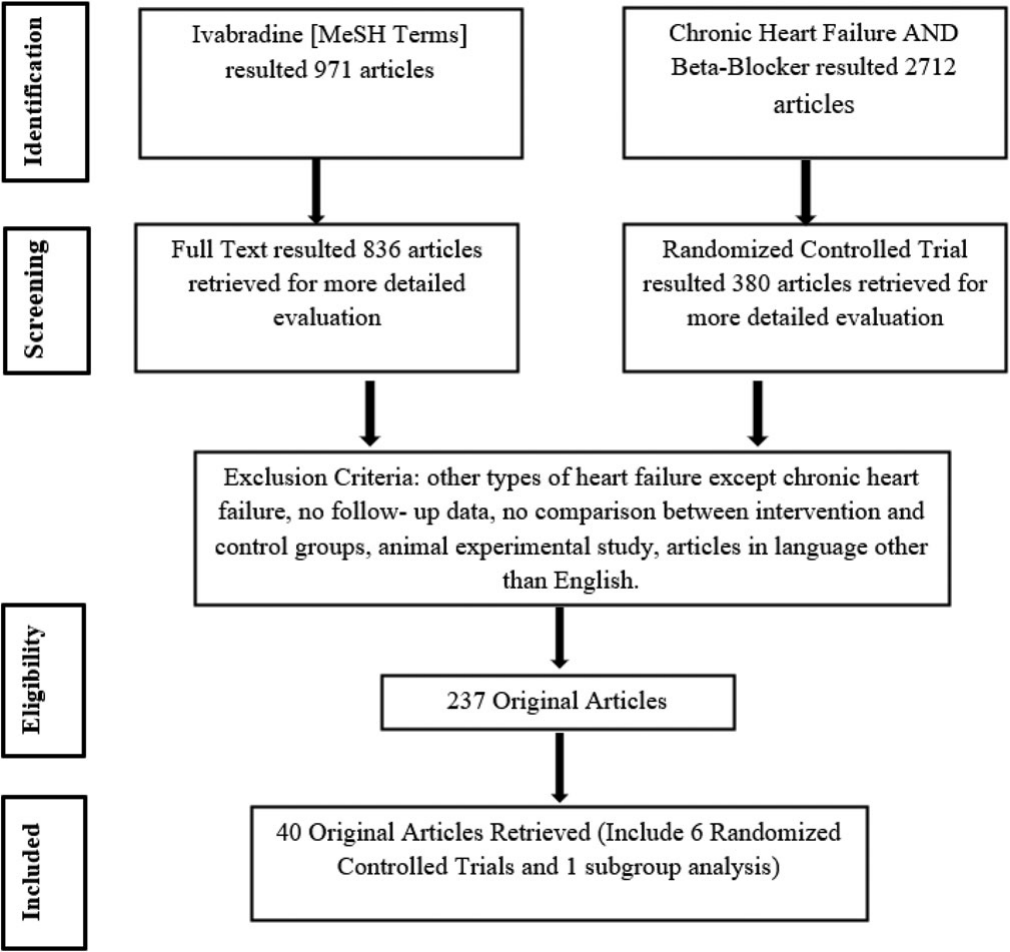
Flow diagram of data collection

We systematically searched PubMed, Medline, Clinical Trials.gov, and The Cochrane Central Register of Controlled Trials for RCTs with search terms Ivabradine (MeSH terms), chronic heart failure and beta‐blocker without any specific time restriction (Figure 1).

### 2.2 Inclusion/exclusion criteria

RCTs with the following inclusion criteria are included^1^: RCT on Ivabradine and published in English^2^; chronic HFrEF^3^; Effect and safety of added Ivabradine (2.5–7.5 mg bid) compared to control group with standard optimal medical treatment, including beta‐blockers, angiotensin‐converting enzyme inhibitors, angiotensin receptor blockers, diuretics, and aldosterone antagonist^4^; Echocardiographic assessment^5^; Exercise tolerance and quality of life (supplement Table 1).

The exclusion criteria were as following: (1) Non‐human studies; (2) Articles in a language other than English; (3) No follow up data; (4) Other types of HF; (5) No comparison between intervention and control groups (Table 1).

**TABLE 1:** Characteristics of included studies

Study or Sub‐study	Method	Participants	Intervention	Outcome	Duration
Tsutsui H et al 2019^22^	Randomized Controlled Trial	254 Japanese patients with age ≥ 20 years old, stable symptomatic chronic HF or NYHA class II‐IV, LVEF≤35%, resting HR ≥75 beats/min in sinus rhythm, received optimal, stable treatment according to Japanese Guideline for Treatment of Chronic Heart Failure and had a history of hospital for worsening HF within the preceding 52 weeks (127 assigned to Ivabradine group, 127 assigned to Placebo)	Ivabradine 5–7.5 mg bid	The primary endpoint was the composite of cardiovascular death or hospital admission for worsening HF.	582 days
Sarullo et al 2010^24^	Randomized Controlled Trial	60 patients with symptoms of heart failure, LVEF≤40%, NYHA classes II to III, sinus rhythm with heart rate at rest>70 beats per minute (bpm), on optimal medical treatment of HF. (30 assigned to Ivabradine group, 30 assigned to Placebo group)	Ivabradine 5 mg bid	Evaluate use of Ivabradine on exercise capacity, gas exchange, functional class, quality of life, and neurohormonal modulation in pts with ischemic CHF	3 months
Mansour et al 2011^21^	Randomized Controlled Trial	53 Idiopathic DCM patients with NYHA class III or IV, LVEF <40%, sinus rhythm, resting heart rate ≥ 70beats/min, on beta‐blocker and ACEI treatment. (30 assigned to Ivabradine group, 23 assigned to Placebo group)	Ivabradine 5–7.5 mg bid	The effect of Ivabradine on symptoms, quality of life, effort tolerance, and echocardiographic parameters in patients with idiopathic DCM with NYHA class III or IV.	3 months
Tsutsui H et al 2016^23^	Randomized controlled trial	126 Japanese patients with age ≥ 20 years old, resting HR ≥75 beats/min in sinus rhythm, stable symptomatic chronic HF of NYHA class II or higher, LVEF≤35%, and under optimal, stable treatment according to Japanese Guideline for Treatment of Chronic Heart Failure (JCS 2010) (84 assigned to Ivabradine group, 42 assigned to Placebo)	Ivabradine 2.5–5 mg bid	Reduction in resting heart rate after 6 weeks treatment.	6 weeks
SHIFT 2010^20^	Randomized controlled trial	6558 patients with symptomatic heart failure and LVEF≤35%, heart rate of 70 bpm or higher (3268 assigned to Ivabradine; 3290 assigned to Placebo group)	Ivabradine 2.5–7.5 mg bid	Cardiovascular death or Hospital readmission for worsening heart failure.	27.8 months
Tardif JC et al 2011 (SHIFT sub‐study)^26^	Randomized controlled Trial	611 Eligible patients in sinus rhythm, resting heart rate ≥ 70 beats/min (bpm), clinically stable for ≥4 weeks, worsening HF within the previous 12 months, and on optimal background therapy for HF including a beta‐blocker. (304 assigned to Ivabradine, 307 assigned to Placebo group)	Ivabradine 2.5–7.5 mg bid	Evaluate the effect of Ivabradine on left ventricular (LV) remodeling in heart failure (HF)	8 months
Volterrani M et al 2011^27^	Randomized controlled trial	80 Eligible patients aged 18 to 90 years, had been diagnosed with HF at least 12 months prior, NYHA Class II‐III, clinically stable for 3 weeks prior to selection or discharged in stable conditions. Patients were receiving optimal background therapy for HF (beta‐blocker, ACEI, ARB, diuretics, aldosterone antagonist) for 3 months. (42 assigned to Ivabradine, 38 assigned to Placebo group)	Ivabradine 2.5–7.5 mg bid	Effect of Ivabradine on the distance covered in 6 minutes walking test (6MWT) and maximal oxygen consumption (MVO_2_) on cardiopulmonary exercise test.	3 months

### 2.3 Outcomes

#### 2.3.1 Therapeutic effect

The main outcomes were the effect of added Ivabradine treatment on chronic HF patient's HR, LVEF, left ventricular remodeling, exercise capacity, and QoL. Evaluation index of reverse remodeling were left ventricular end‐systolic diameter (LVESD), left ventricular end‐diastolic diameter (LVEDD), left ventricular end‐diastolic volume (LVEDV), and left ventricular end‐systolic volume (LVESV). Assessment for exercise capacity was measured with exercise duration, meanwhile, Minnesota Living with Heart Failure (MLWHF) questionnaire were used to detect an improvement in the QoL of HF patients. (supplement Tables 2 and 3).

#### 2.3.2 Safety

Adverse events include cardiovascular mortality, rehospitalization for worsening HF, asymptomatic bradycardia, and visual disturbances were recorded in Ivabradine and placebo groups.

### 2.4 Data analysis

Data analysis was done by using RevMan 5.4, dichotomous data are reported by using Mantel–Haenszel statistical method, fixed/random effects analysis model, risk ratio effect measure with 95% CIs, while Continuous variables are evaluated using mean differences (MD) with 95% CIs. Effect model was used in data analysis depends on the degree of heterogeneity and *P*‐value, a fixed‐effect model was used if I^2^ < 50% and *P*‐value >.10, while random effect model preferred in high heterogeneity I^2^ > 50% and low *P*‐value <.10. If heterogeneity was detected, subgroup analyses to explore the source of heterogeneity will be conducted. Meanwhile, a sensitivity analysis to evaluate robustness of the outcomes was done by removing the study with high risk or unclear risk of selection bias.

### 2.5 Assessment risk of bias and quality of studies

The Cochrane risk of bias domains were used to analyze the bias ratings of each study. The selection of domains includes random sequence generation, allocation concealment, blinding of participants and personnel, blinding of outcome assessment, incomplete outcome data, selective reporting, and other bias. Ratings of bias are divided into low risk, unclear risk, and high risk. Quality of evidence extracted by two independent investigators (Richard Bryan and Bi Huang), where the disagreement about inclusion data will be settled by a third investigator (Gang Liu) through a discussion and consensus.

## 3 RESULTS

In total, six RCTs and one subgroup analysis with 7074 participants (3523 in the placebo group, 3551 in the Ivabradine group) were enrolled in this meta‐analysis. The main outcomes of this study were the effect of Ivabradine therapeutic on HR, LVEF, exercise capacity, QoL, and left ventricular remodeling. The high heterogeneity presented might be attributed to a distinct measurement index, insufficient number of studies on preferred outcomes, and different baseline characteristics of represented studies, such as sample size, age, gender, and follow‐up time.

### 3.1 Effect of ivabradine on main outcomes

**FIGURE 2:**
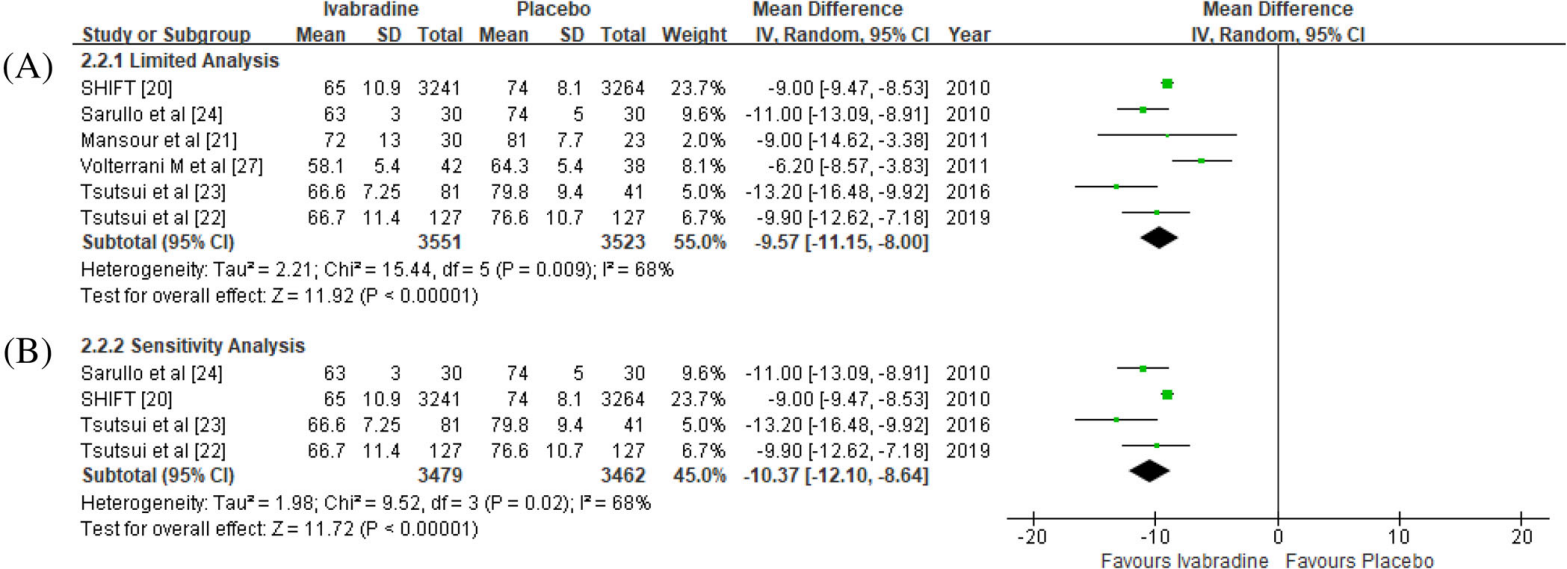
Effect of Ivabradine versus placebo on heart rate reduction. (A) limited analysis; (B) sensitivity analysis

Six RCTs included in our study showed that Ivabradine added to the standard HF treatment was associated with a better optimization for resting HR reduction in chronic HFrEF patients (MD = −9.57; 95% CI ‐11.15, −8.00) compared to the placebo group.^20^, ^21^, ^22^, ^23^, ^24^, ^27^ Sensitivity analysis with removal of high risk or unclear risk selection bias study showed consistent findings on the effect of Ivabradine for further HR reduction (MD = −10.37; 95%CI ‐12.10, −8.64) (Figure 2).

**FIGURE 3:**
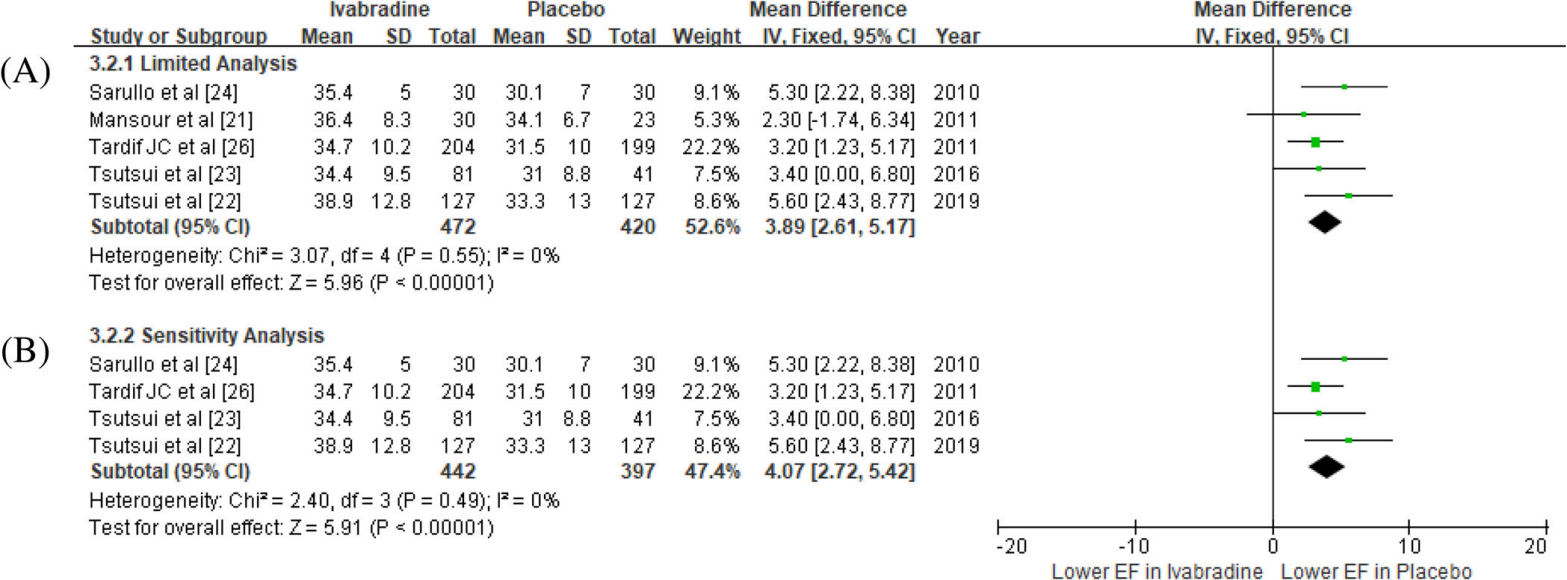
Effect of added Ivabradine compared with placebo in LV ejection fraction. (A) limited analysis; (B) sensitivity analysis

Five RCTs (420 in Placebo, 472 in Ivabradine) demonstrated treatment with added Ivabradine significantly increased LVEF in the chronic HFrEF patients (MD = 3.89; 95% CI 2.61, 5.17).^21^, ^22^, ^23^, ^24^, ^25^, ^26^ (Figure 3). A sensitivity analysis maintained the effect of LVEF improvement (MD = 4.07; 95%CI 2.72, 5.42).

Three echocardiographic studies of chronic HFrEF (252 in Placebo, 264 in Ivabradine) were enrolled to observe the possibility of Ivabradine for left ventricular remodeling. Analysis was done by dividing parameter index into four groups including LVEDD,^21^, ^24^ LVESD,^21^, ^24^ LVEDV,^21^, ^24^, ^26^ and LVESV.^21^, ^24^, ^26^ Based on these four parameters, our study showed added Ivabradine on the standard HF therapy distinctly improved echocardiographic parameters, with only LVEDV not achieving statistical significance (LVESD; MD = −3.73; 95% CI ‐4.25, −3.21, LVESV; MD = −17.00, 95%CI ‐29.65, −4.35, LVEDD; MD = −1.43, 95%CI ‐2.78, −0.08, LVEDV; MD = −14.75, 95%CI ‐34.36, 4.87) (supplement Figure 1). A sensitivity analysis was performed on LVEDV and LVESV, with LVESV maintained the positive outcome, while LVEDV displayed no significant differences (MD = −5.77;95%CI ‐11.94, 0.39; MD = −11.48; 95%CI ‐17.10, −5.86, respectively).

Exercise capacity between optimal HF treatment compared to added Ivabradine treatment was measured by total exercise duration.^21^, ^24^ It revealed added Ivabradine had better exercise tolerance than placebo group (MD = 8.52; 95% CI 0.09, 16.94) (supplement Figure 2).

Two studies (53 in Placebo, 60 in Ivabradine) were enrolled to observe the improvement of QoL in chronic HFrEF patients. It indicated that added Ivabradine treatment had no significant effect on MLWHF score (MD = 0.65; 95%CI ‐10.52, 11.82) (supplement Figure 3).

### 3.2 Adverse events

**FIGURE 4:**
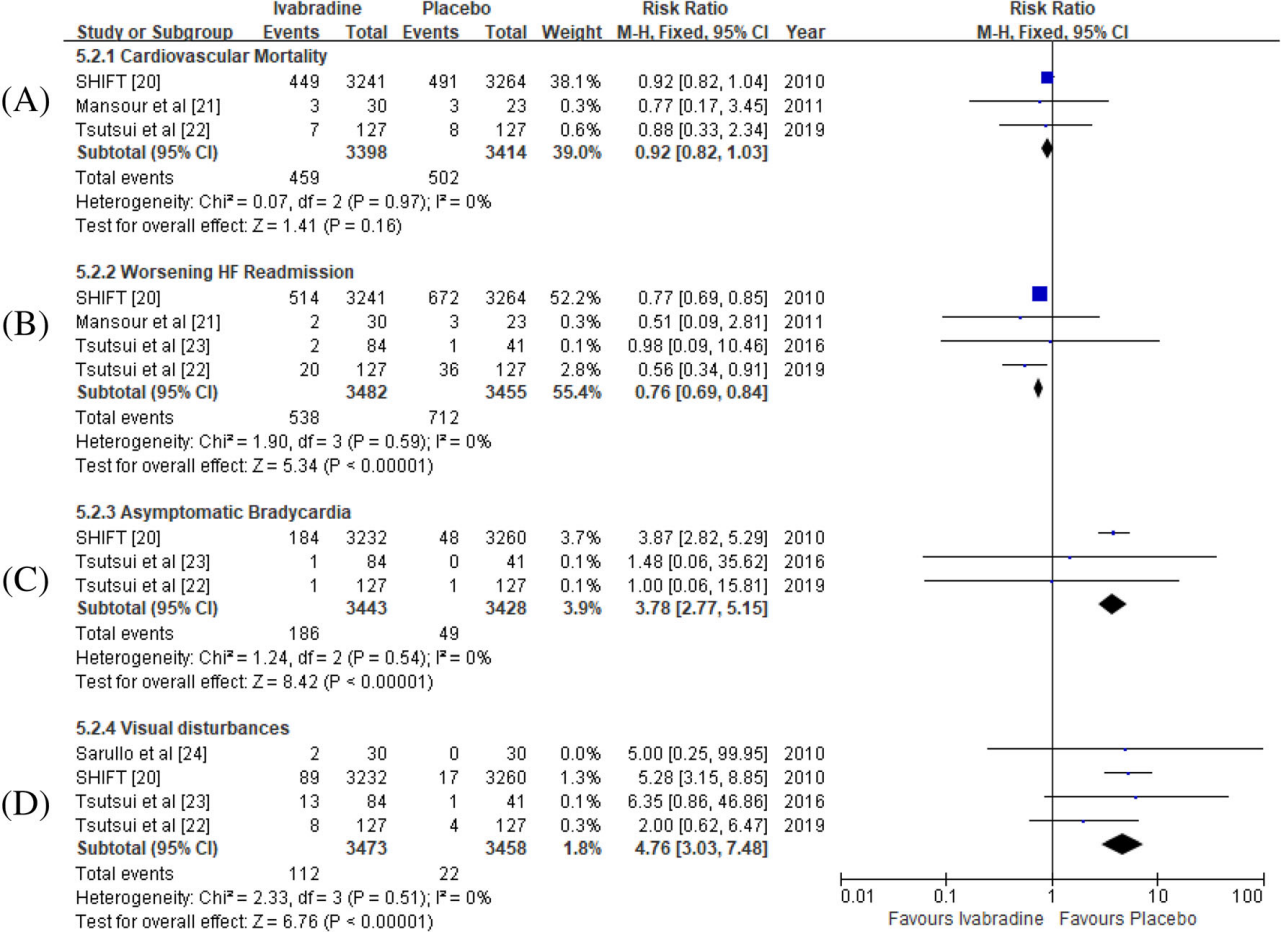
Adverse events in Ivabradine group versus placebo group post‐treatment. (A) cardiaovascular mortality; (B) hospital re‐admission for worsening heart failure; (C) visual disturbances; D: asymptomatic bradycardia

The therapeutic safety evaluation index of Ivabradine was analyzed by using risk ratio, and fixed/random effect model. Included side effects were cardiovascular death, rehospitalization for worsening HF, visual disturbances, and asymptomatic bradycardia. Despite that multiple large studies showed inconsistency results on HF rehospitalization,^19^, ^20^, ^28^ our meta‐analysis showed significant reduction for worsening HF rehospitalization with Ivabradine treatment (RR = 0.76; 95%CI 0.69, 0.84), no significant differences for cardiovascular mortality (RR = 0.92; 95%CI 0.82, 1.03). Visual disturbances and asymptomatic bradycardia events were significantly increased in Ivabradine group (RR = 4.76; 95%CI 3.03, 7.48; RR = 3.78; 95%CI 2.77, 5.15) (Figure 4).

### 3.3 Risk of bias and quality assessment

Based on the Cochrane Collaboration for risk of bias assessment criteria, enrolled studies presented various risk of bias. Moreover, the assessment of other possible bias is uncertain due to insufficient information from respective studies (supplement Figure 4).

### 3.4 Investigation of heterogeneity

High heterogeneity investigation in the resting HR was stratified into two subgroups based on age and follow‐up duration. First, included studies were grouped based on the age < 60 years old^21^, ^23^, ^24^ and ≥ 60 years old,^20^, ^22^, ^27^ and revealed that the results were consistent in the two different age groups (MD = −11.40; 95%CI ‐13.08, −9.72; MD = −8.46; 95%CI ‐10.24, −6.68, respectively). However, substantial heterogeneity presented in the subgroup of ≥60 years old (I^2^ = 65%), but not in the <60 years old group (I^2^ = 0%), thus indicating aging was one of the reasons for the high heterogeneity.

Second, subgroup analysis was performed based on the duration of Ivabradine treatment for HR reduction with distinction <6 months^21^, ^23^, ^24^, ^27^ and ≥ 6 months.^20^, ^22^ In the <6 months subgroup, four RCTs demonstrated high heterogeneity (MD ‐9.84; 95%CI ‐13.11, −6.58; I^2^ 79%), nevertheless in ≥6 months group 2 RCTs showed no heterogeneity (MD ‐9.03; 95%CI ‐9.49, −8.57; I^2^ 0%).

In the present study, high heterogeneity also existed in the other outcomes, such as in the left ventricular remodeling indexes, exercise capacity, and QoL. As for the LV remodeling parameters, after conducting a sensitivity analysis, low heterogeneity was displayed on LVEDV and LVESV. Meanwhile, owing to an insufficient number of studies, subgroup analyses for exercise capacity and QoL was unable to be conducted.

## 4 DISCUSSION

The present meta‐analysis demonstrated apart from several adverse events mentioned, added Ivabradine treatment was not only safe, but also effective for HR reduction, improvement of cardiac function, and better exercise tolerance in patients with chronic HFrEF. To the best of our knowledge, this meta‐analysis is among the few meta‐analyses to discuss the effect of Ivabradine in chronic HFrEF patients.

In terms of the efficiency of Ivabradine, the present meta‐analysis complies with SHIFT^20^ study and Pei, et al^29^ s study in which Ivabradine could reduce the risk of worsening HF readmission (HR = 0.74; 95%CI 0.66, 0.83, RR = 0.91; 95%CI 0.85, 0.97, respectively). However, the finding was not confirmed by other three meta‐analyses in which Koroma, et al,^30^ Benstoem C, et al,^31^ and Anantha, et al^32^ meta‐analyses demonstrated no significant reduction in worsening HF rehospitalization. The inconsistent conclusion was possibly due to the different inclusion criteria in a respective study such as in Anantha, et al^32^ meta‐analysis which included one acute HF study^33^ and one stable angina study^34^ in addition to HF studies, while our meta‐analysis only included chronic HFrEF studies. Moreover, although several meta‐analyses including ours reached similar conclusions such as the improvement of LVEF after addition of Ivabradine to standard therapy, however, our study also had some distinctive features compared with these meta‐analyses. First, the most important superiority is that the participants in our meta‐analysis were pure patients with chronic HFrEF. Second, we reconfirmed the neutral effect of Ivabradine on cardiovascular mortality in chronic HFrEF patients, although it could improve LVEF. Moreover, we systematically evaluated the safety and efficiency with clinical events, echocardiographic parameters, exercise tolerance and QoL scores while other meta‐analyses mainly focused on some specific issues such as HR changes,^31^, ^32^ cardiac remodeling,^35^ diastolic dysfunction^30^ or the safety of Ivabradine.^29^, ^31^

Ivabradine lead to improvement in HR and LVEF was mainly attributed to the fact that the combination with beta‐blocker produces a significant reduction in the resting HR. Tsutsui, et al^23^ found that high‐dose (7.5 mg bid) of Ivabradine had a greater reduction in the HR change compared to low‐dose (2.5 mg bid) of Ivabradine (84.0 ± 7.5 to 67.1 ± 8.0, 81.2 ± 7.0 to 66.0 ± 9.0, respectively). In fact, due to the HR reduction mechanism, Ivabradine at first was recognized as an anti‐anginal agent and several studies also enrolled patients with stable coronary artery disease as the main inclusion criteria such as the BEAUTIFUL^19^ and SIGNIFY^28^ studies. However, these studies demonstrated no significant improvement in terms of all‐cause death, readmission to hospital for worsening HF, and cardiovascular death. This finding is supported by Maagaard M, et al's meta‐analysis.^36^ Our present meta‐analysis had superiority with only RCTs with chronic HFrEF patients and could reach reliable conclusions.

Association between dosage of Ivabradine and LVEF improvement was also found in some studies. Tsutsui, et al^23^ demonstrated higher dosage of Ivabradine was positively associated with better improvement in LVEF (5 mg bid, 28.6 ± 4.8 to 35.0 ± 10.4; 2.5 mg bid. 28.4 ± 5.6 to 33.8 ± 8.7). This finding is consistent with Raja, et al's study^37^ in which HR≤70 beats per minute had higher LVEF compared with that of HR > 70 beats per minute (30.4 ± 3.8%, 27.6 ± 3.6, respectively). These studies indicated that optimal control of HR to less than 70 beats per minute with Ivabradine was more beneficial to the LVEF improvement.

Moreover, the improvement of exercise capacity is beneficial from the combination of beta‐blocker and Ivabradine which increase the beta‐blocker non‐selective effects on the alpha‐adrenergic receptor, thus affect muscle‐skeletal dilatation during exercise^.^ ^38^ Furthermore, Ivabradine has been shown to preserve exercise‐induced vasodilation, increase muscular blood flow during exercise, and improvement on peripheral blood flow.^38^ Volterrani M, et al^27^ assessed the effect of Ivabradine on chronic HF patient's exercise tolerance by using 6 minutes walking test, in which the Ivabradine group demonstrated greater improvement than placebo group (Ivabradine; 358.2 ± 107.6 to 453.1 ± 87.4; Placebo; 379.0 ± 96.3 to 435.7 ± 121.3). Our meta‐analysis is also in compliance with Koroma, et al^30^ finding which Ivabradine therapy increased exercise tolerance (6MWT; SMD = −1.01; 95%CI ‐0.59, −0.06).

Most studies inferred the observed effect of Ivabradine on QoL improvement was likely to be related to exercise capacity improvement,^38^ although our present meta‐analysis showed no significant difference in QoL improvement between the two groups. However, we found two studies displayed a positive association between Ivabradine with chronic HF patients' QoL improvement. Ekman I, et al.^39^ conducted a SHIFT trial sub‐study on the association of Ivabradine with QoL improvement by using the Kansas City Cardiomyopathy Questionnaire (KCCQ) and they found that added Ivabradine treatment demonstrated better KCCQ overall summary score (Placebo 65.3 ± 19.8 to 69.6 ± 16.7; Ivabradine 65.2 ± 20 to 71.9 ± 17.3, *p* < .001). Moreover, Volterrani M, et al^27^ observed quality of life improvement by using MacNew QLMI (quality of life after myocardial infarction) questionnaire in patients with HF complicating myocardial infarction, and they found Ivabradine group (4.7 ± 0.8–6.1 ± 0.6) showed higher QoL improvement than placebo group (4.6 ± 0.8–4.1 ± 0.6).

Ivabradine was thought to have no relation with renin angiotensin aldosterone (RAA) system and the sympathetic nervous system, therefore, theoretically, Ivabradine does not affect cardiac reverse remodeling. However, our meta‐analysis included two RCTs and one subgroup analysis favors adding Ivabradine for reverse remodeling in chronic HFrEF patients.^21^, ^24^, ^26^ This finding was in accordance with Wan H, et al's study,^35^ which demonstrated Ivabradine had a positive association with reverse cardiac remodeling (LVESVI; MD = −7.30; 95%CI ‐12.94, −1.66; LVEDVI; MD = −7.27; 95%CI ‐14.04, −0.50). Several studies hypothesized the potential mechanisms associated with Ivabradine improving cardiac remodeling were the modification in cardiac myocyte function, optimization of energy consumption, improvement of endothelial function, a reversal in electrophysiological changes, to the extent of reduction of RAAS stimulation and sympathetic drive etc.^40^ However, due to insufficient large RCTs, we still cannot conclude whether Ivabradine can absolutely improve left ventricular remodeling or not, also to what extent Ivabradine can improve the outcome, therefore more large studies are still needed to clarify the role that Ivabradine played in cardiac remodeling.

## 5 LIMITATIONS

There are several unavoidable limitations in this study. First, although we included six RCTs and one subgroup analysis associated with Ivabradine efficiency and safety in patients with chronic HFrEF, however, the high heterogeneity and the numbers of patients included to each study contributed to the statistical analysis and conclusion. Second, different parameter indices associated with LV remodeling, exercise capacity, and quality of life were used in different studies, thus limiting the analysis with the same parameters and also the final conclusion. Third, echocardiographic indexes as ventricular remodeling indicators are easily affected by afterload and preload, thus careful and repeated measurement of cardiac echocardiography is necessary. Fourth, most of the included studies only provided average doses of Ivabradine and therefore we could not get the dose‐effect relationship. Therefore, more large‐scale studies are still needed to elucidate the association of Ivabradine with the outcome in patients with chronic HFrEF.

## 6 CONCLUSIONS

Addition of Ivabradine to standard HF therapy is associated with cardiac function improvement, reduction on worsening HF readmission, greater HR reduction, and better exercise capacity in chronic HFrEF patients, although it cannot reduce cardiovascular mortality or improve the quality of life.

**Abbreviations**
6MWT: 6 minutes walking test
ACEI: angiotensin converting enzyme inhibitor
AECOPD: acute exacerbation chronic obstructive pulmonary disease
ARB: angiotensin receptor blocker
BEAUTIFUL: effects of ivabradine in patients with stable coronary artery disease and left ventricular systolic dysfunction
BPM: beats per minute
CHF: chronic heart failure
CI: confidence interval
CQMU: ChongQing Medical University
CVD: cardiovascular death
FDA: Food and Drug Administration
HF: heart failure
HFrEF: heart failure reduced ejection fraction
HR: heart rate
KCCQ: Kansas City Cardiomyopathy Questionnaire
LVEDD: left ventricular end‐diastolic diameter
LVEDV: left ventricular end‐diastolic volume
LVEF: left ventricular ejection fraction
LVESD: left ventricular end‐systolic diameter
LVESV: left ventricular end‐systolic volume
LVSD: left ventricular systolic dysfunction
MD: mean differences
MeSH: medical subject heading
MLWFH: Minnesota living with heart failure
PRISMA: Preferred Reporting Items for Systematic Review and Meta‐Analysis
QLMI: quality of life after myocardial infarction
QoL: quality of life
RAAS: renin angiotensin aldosterone system
RCT: randomized controlled trials
RevMan: review manager
RR: risk ratios
SHIFT: effects of ivabradine on cardiovascular events in patients with moderate to severe chronic heart failure and left ventricular systolic dysfunction

## CONFLICT OF INTEREST

The authors declare no competing interests.

## Supporting information


**Appendix** S1. Supporting information

**Appendix** S2. Supporting information

**Figure S1** Ivabradine vs Placebo on Echocardiographic studies. A: Left Ventricular End‐Diastolic Diameter (LVEDD); B: Left Ventricular Systolic Diameter (LVESD); C: Left Ventricular End‐Diastolic Volume (LVEDV) limited and sensitivity analysis; D: Left Ventricular End‐Systolic Volume (LVESV) limited and senstivity analysis.
Supplement Figure 2. Ivabradine vs Placebo on Exercise Capacity Improvement.
Supplement Figure 3. Ivabradine versus Placebo in Minnesota Living with Heart Failure (MLWHF) questionnaire
Supplement Figure 4. Risk of Bias Summary for Included Studies

**Table S1** Standard Heart Failure Treatment at Randomization
Supplement Table 2. Baseline Characteristics of Included Studies
Supplement Table 3. Post‐Follow up Outcomes

## Data Availability

The data that supports the findings of this study are available in the supplementary material of this article.
