# Supplementary material for: Impact of ivabradine on the cardiac function of chronic heart failure reduced ejection fraction: Meta‐analysis of randomized controlled trials
Source: Clin Cardiol. 2021 Feb 27;44(4):463–71. doi: 10.1002/clc.23581 (PMC8027585; doi:10.1002/clc.23581)
Supplement: Supplementary file 1 — Appendix S1. Supporting information [file CLC-44-463-s001.docx]

**Impact of Ivabradine on The Cardiac Function of Chronic Heart Failure Patients: Meta-analysis of Randomized Controlled Trials**

*Richard BRYAN MBBS, Bi HUANG MD, Gang LIU MD, Yuan YANG MD and Suxin LUO MD*

Funding/Support: Supported by National Key R&D Program of China (2018YFC1311400, 2018YFC1311404)

**Objectives**

The aim of this meta-analysis was to elucidate the effect of Ivabradine in chronic heart failure reduced ejection fraction (HFrEF) patients, especially the cardiac function. Specifically, this study was designed to observe the effect of standard heart failure treatment against added Ivabradine therapy on cardiac function, reverse remodeling, and rehospitalization due to worsening HF.

**Searches**

We will search PubMed，Medline，Clinical Trials.gov, and the Cochrane Central Register of Controlled Trials for randomized controlled trials with search terms Ivabradine [MeSH Terms], beta-blocker and chronic heart failure without any specific time restriction.

**Types of study to be included**

All randomized clinical trials about a comparison of added Ivabradine treatment versus placebo on chronic HFrEF patients regardless of study method, duration of follow up and sample of the study. Studies with a large number of missing data, no follow-up time, non-human studies, other types of heart failure except chronic HFrEF, other languages than English, and do not have a clear measure of outcome interest will be excluded from the study.

**Condition or domain being studied**

Heart failure is known as progressive disease and terminal stage of multiple cardiovascular diseases, risk of developing heart failure increase with aging, which is why usually it affects older people. Impaired systolic or/and diastolic function of the heart will lead into cardiac remodeling, and several complications such as cardiac arrhythmia, myocardial infarction, kidney damage, etc. Thus, explained why chronic heart failure has a poor prognosis, high mortality rate, as well as heavy economic burden.

According to the current guideline, heart failure treatment mainly are beta-blockers, angiotensin converting enzyme inhibitor (ACEI) or angiotensin receptor blocker (ARB), loop diuretics, and aldosterone antagonist. Resting heart rate control is important in heart failure patients, as several studies have emphasized increased resting heart rate in HF patients associated with a higher mortality rate and higher incidence of heart failure.

Beta-blocker is the main therapy for heart rate reduction; however, it may have several limitations and undesirable side effects, thus a pure bradycardic agent called Ivabradine which selectively inhibit If current in sinoatrial node has been developed. Ivabradine has been approved with indications of heart failure reduced ejection fraction (HFrEF) and sinus rhythm ≥70 beats per minute on a maximal dosage of beta-blocker or when beta-blocker is contraindicated. Although patients in clinical trials have been reported with some prominent undesirable effects such as atrial fibrillation, visual disturbances, and symptomatic bradycardia, however in this study we attempted to find the possibility of Ivabradine for cardiac function improvement.

**Intervention**

Added Ivabradine treatment will be the intervention group. We will include all chronic heart failure reduced ejection fraction (HFrEF) randomized controlled trials regardless of the size of sample, dosage, frequency, and duration of treatment.

**Control**

Standard heart failure treatment will be the control group.

**Main Outcomes**

Heart Rate, Ejection Fraction, Ventricular Remodeling, Exercise Capacity, and Quality of Life.

**Additional Outcomes**

Adverse effects of Ivabradine, they are cardiac mortality, rehospitalization for worsening heart failure, asymptomatic bradycardia, and visual disturbances.

**Data Extraction (selection and coding)**

2 Independent researchers R.B and B.H will extract the following information from included studies:

1. General information of each studies (date of data extraction).
2. Author, Article Title, Citation, Journal publication, abstract, source of funding, record number of identify study.
3. Objectives of included studies which correlate with our objectives (study design, aim of study, inclusion/exclusion criteria, randomized controlled trials).
4. Baseline characteristics of included studies population (age, sex, number of participants in each characteristics category of intervention and control group.
5. Dose and route of administration, duration of treatment, outcome results after follow up, analysis of the outcome data.
6. Statistical analysis methods used.
7. Summary outcome of data, for example dichotomous data are reported using Mantel-Haenszel statistical method, fixed/random effects analysis model, risk ratio effect measure with 95% CIs, while Continuous variables are evaluated using mean differences (MD) with 95% CIs. Summary for each pre-specified outcome (number of participants, number of withdrawals, lost to follow up, exclusions).

**Risk of Bias Assessment**

2 Independent reviewers (RB and BH) will assess the risk of bias of each included study, any disagreement will be solved by third reviewer (GL). We used the Cochrane risk of bias domains to analyze bias ratings of each studies. The selection of domains includes random sequence generation, allocation concealment, blinding of participants and personnel, blinding of outcome assessment, incomplete outcome data, selective reporting, and other bias. Ratings of bias divided into low risk, unclear risk, and high risk.

1. Random Sequence Generation: low risk of bias (inadequate method for example random numbers or tables); uncertain risk of bias (included study didn’t mention randomization method); high risk of bias (possible confounding exist).
2. Allocation Concealment: low risk of bias (less or no bias on the final results); uncertain risk of bias (insufficient information and method on possibility of bias); high risk of bias (pharmacy controlled, open random allocation).
3. Blinding of Participants and Personnel: low risk of bias (adequate blinding of participants); unclear risk of bias (insufficient information whether blinding may influence the effect of intervention); high risk of bias (no blinding or incomplete blinding, and estimation of outcome affected by lack of blinding).
4. Blinding Outcome Assessment: low risk of bias (outcome not influenced by blinding); unclear risk of bias (unclear whether outcome influenced by blinding or not); high risk of bias (no blinding or incomplete blinding, outcome affected by lack of blinding).
5. Incomplete Outcome Data: low risk of bias (missing data would not induce bias, proper methods handle the missing data); unclear risk of bias (lack of information whether incomplete data may affect the outcome or not); high risk of bias (method to handle missing data insufficient, participants drop out due to different reasons).
6. Selective Reporting: low risk of bias (trial protocol exist, prespecified outcomes reported); unclear risk of bias (insufficient information whether selective outcome affected by observed effect or not); high risk of bias (not all prespecified outcomes reported).
7. Other Bias (Source of Funding Bias): low risk of bias (support/funding did not come from drug manufacturer); unclear risk of bias (source of funding was not clear); high risk of bias (supported by drug manufacturer).

**Assessment Quality of Studies**

2 Independent reviewers (RB and BH) will assess the quality of outcomes, any disagreement will be resolved by a third independent reviewer (L.G).

**Data Synthesis**

Data analysis was done by using RevMan 5.4, dichotomous data are reported using Mantel-Haenszel statistical method, fixed/random effects analysis model, risk ratio effect measure with 95% CIs, while Continuous variables are evaluated using mean differences (MD) with 95% CIs. Effect model was used in data analysis depends on the degree of heterogeneity and P value, fixed effect model was used if I^2^ <50% and P value >0.10, while random effect model preferred in high heterogeneity I^2^ >50% and low P value <0.10. High heterogeneity will be investigated by conducting subgroup analysis. Sensitivity analysis will be done by removing high risk or unclear risk of selection bias.

**Contact Details for Further Information**

Dr Bryan Richard

1400885752@qq.com

**Affiliation of the review**

None

**Review team members**

Dr Bryan Richard. The First Affiliated Hospital of Chongqing Medical University

Dr Liu Gang. The First Affiliated Hospital of Chongqing Medical University

Dr Huang Bi. The First Affiliated Hospital of Chongqing Medical University

Dr Yang Yuan. The First Affiliated Hospital of Chongqing Medical University

Dr Luo Suxin. The First Affiliated Hospital of Chongqing Medical University

**Anticipated Start Date**

28 August 2020

**Anticipated Completion Date**

30 November 2020

**Funding/Support**

Supported by National Key R&D Program of China (2018YFC1311400, 2018YFC1311404)

**Conflict of Interest**

None

**Language**

English

**Country**

China
