# Supplementary figures and images for: Impact of ivabradine on the cardiac function of chronic heart failure reduced ejection fraction: Meta‐analysis of randomized controlled trials
Source: Clin Cardiol. 2021 Feb 27;44(4):463–71. doi: 10.1002/clc.23581 (PMC8027585; doi:10.1002/clc.23581)

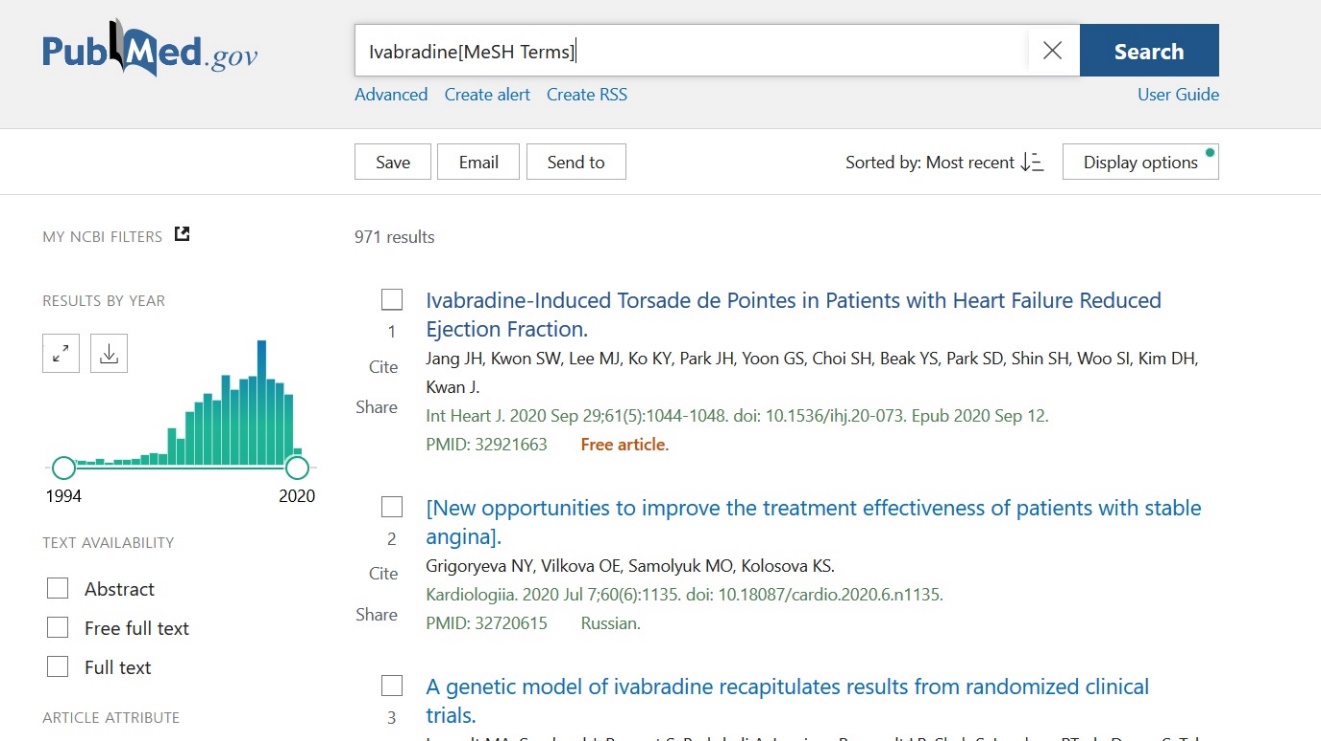


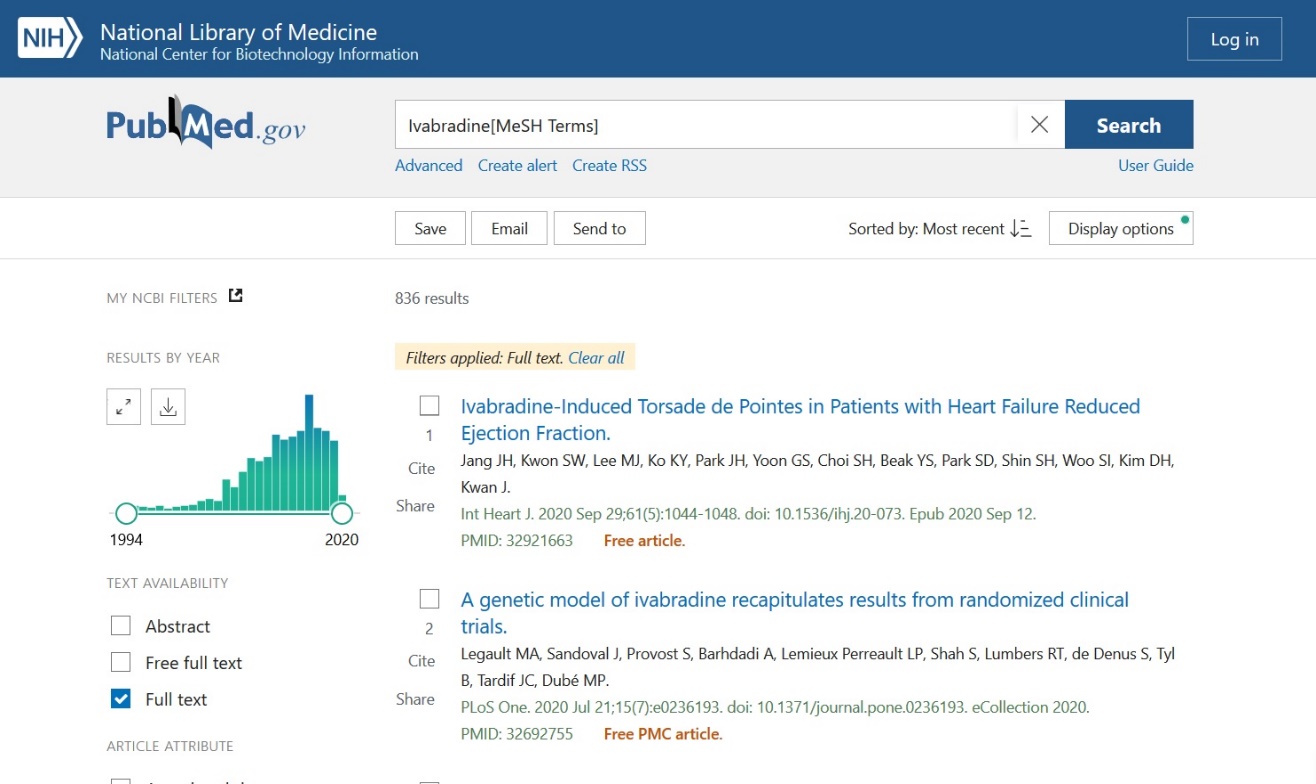


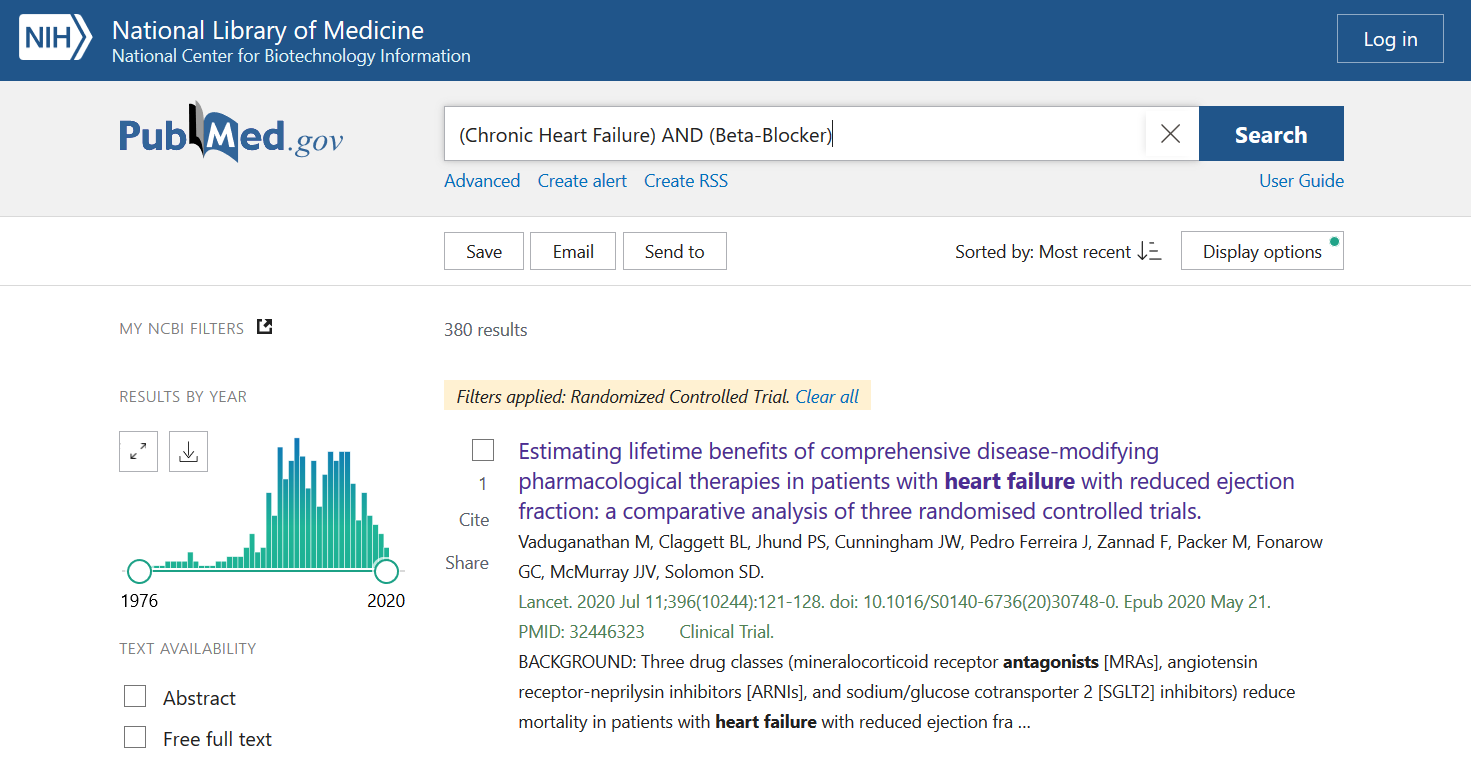

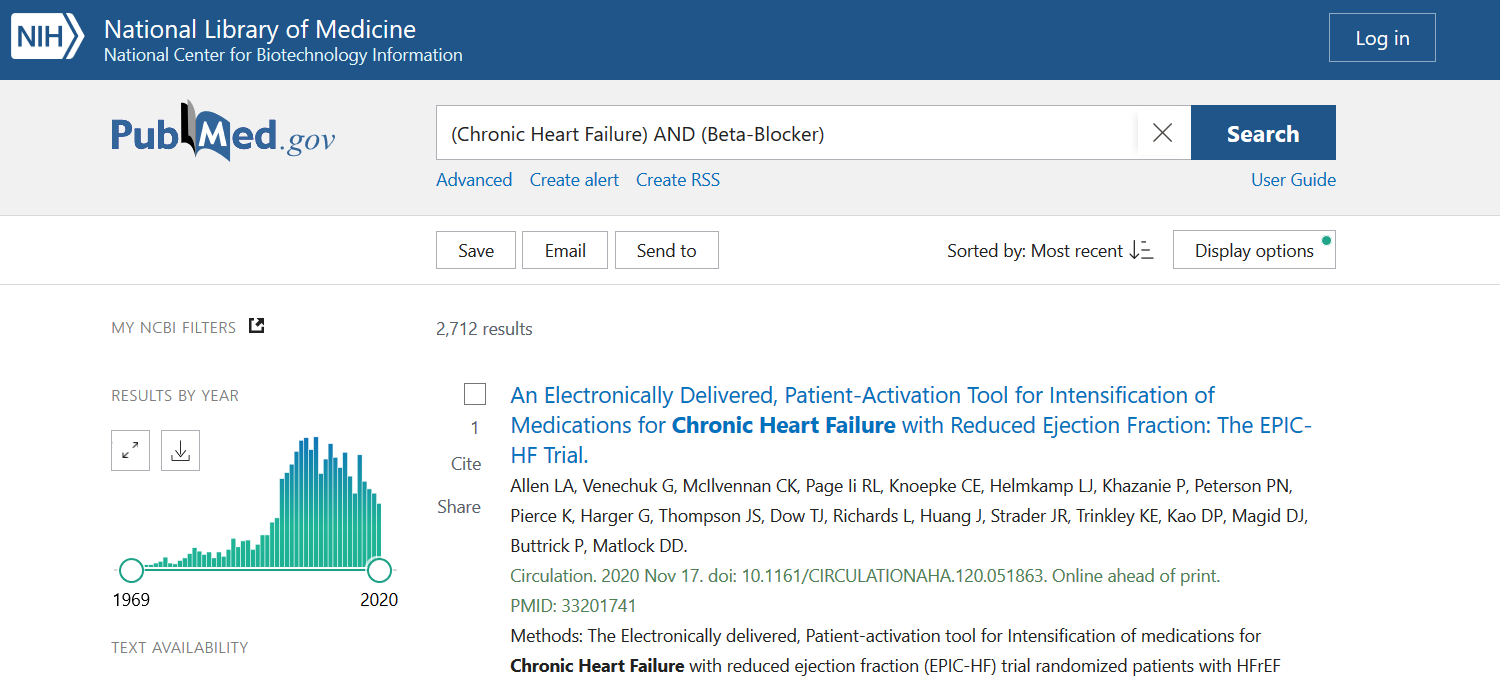

Supplement: Supplementary file 2 — Appendix S2. Supporting information [file CLC-44-463-s003.docx]
