# Supplementary material for: Impact of ivabradine on the cardiac function of chronic heart failure reduced ejection fraction: Meta‐analysis of randomized controlled trials
Source: Clin Cardiol. 2021 Feb 27;44(4):463–71. doi: 10.1002/clc.23581 (PMC8027585; doi:10.1002/clc.23581)
Supplement: Supplementary file 3 — Figure S1 Ivabradine vs Placebo on Echocardiographic studies. A: Left Ventricular End‐Diastolic Diameter (LVEDD); B: Left Ventricular Systolic Diameter (LVESD); C: Left Ventricular End‐Diastolic Volume (LVEDV) limited and sensitivity analysis; D: Left Ventricular End‐Systolic Volume (LVESV) limited and senstivity analysis. Supplement Figure 2. Ivabradine vs Placebo on Exercise Capacity Improvement. Supplement Figure 3. Ivabradine versus Placebo in Minnesota Living with Heart Failure (MLWHF) questionnaire Supplement Figure 4. Risk of Bias Summary for Included Studies [file CLC-44-463-s004.docx]

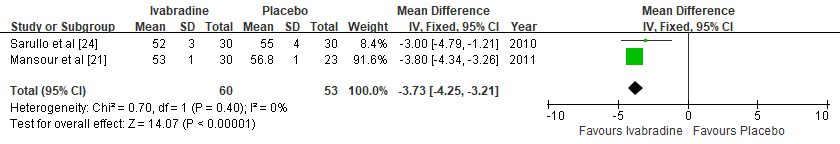

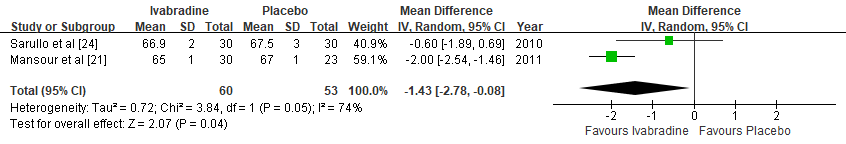


B

A


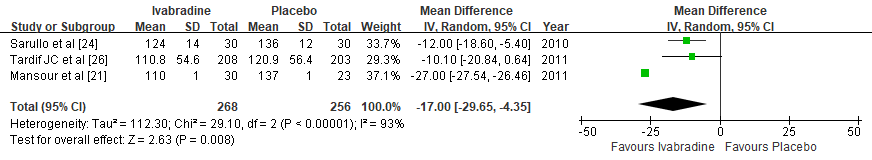

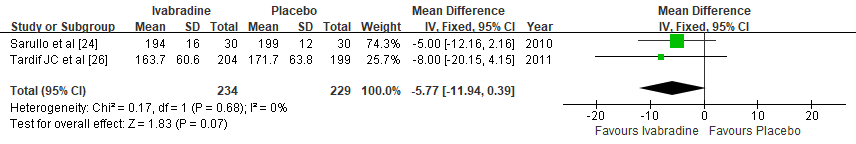

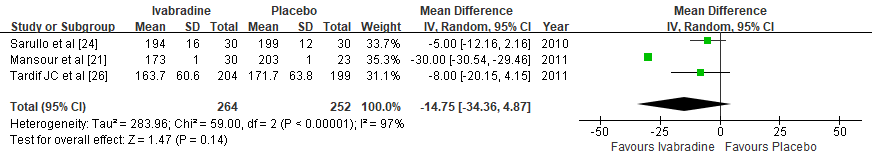


C


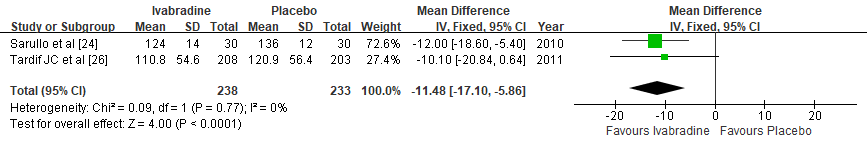


D

Supplement Figure 1. Ivabradine vs Placebo on Echocardiographic studies. A: Left Ventricular End-Diastolic Diameter (LVEDD); B: Left Ventricular Systolic Diameter (LVESD); C: Left Ventricular End-Diastolic Volume (LVEDV) limited and sensitivity analysis; D: Left Ventricular End-Systolic Volume (LVESV) limited and senstivity analysis.


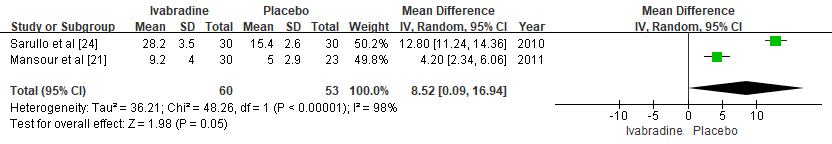
Supplement Figure 2. Ivabradine vs Placebo on Exercise Capacity Improvement.


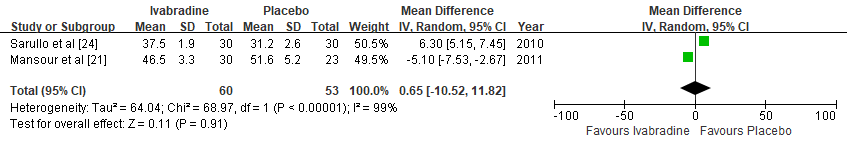
Supplement Figure 3. Ivabradine versus Placebo in Minnesota Living with Heart Failure (MLWHF) questionnaire


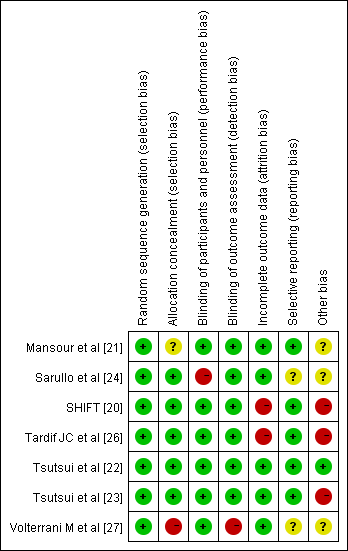
Supplement Figure 4. Risk of Bias Summary for Included Studies
