# Supplementary material for: Impact of ivabradine on the cardiac function of chronic heart failure reduced ejection fraction: Meta‐analysis of randomized controlled trials
Source: Clin Cardiol. 2021 Feb 27;44(4):463–71. doi: 10.1002/clc.23581 (PMC8027585; doi:10.1002/clc.23581)
Supplement: Supplementary file 4 — Table S1 Standard Heart Failure Treatment at Randomization Supplement Table 2. Baseline Characteristics of Included Studies Supplement Table 3. Post‐Follow up Outcomes [file CLC-44-463-s002.docx]

Supplement Table 1. Standard Heart Failure Treatment at Randomization

| Treatments at randomization | **Tsutsui H et al 2019** | **Sarullo et al 2010** | **Mansour et al 2011** | **Tsutsui H 2016** | **SHIFT 2010** | **Tardif JC 2011** | **Volterrani M et al 2011** |
| --- | --- | --- | --- | --- | --- | --- | --- |
| ***β-blocker*** |  |  |  |  |  |  |  |
| Placebo | 120 (94.5%) | 19 (63.3%) | 23 (100%) | 39 (92.9%) | 2923 (90%) | 281 (92%) | 22 (58%) |
| Ivabradine | 122 (96.1%) | 17 (56.6%) | 30 (100%) | 78 (92.9%) | 2897 (89%) | 281 (92%) | 23 (55%) |
| ***ACE Inhibitor*** |  |  |  |  |  |  |  |
| Placebo | 67 (52.8%) | 26 (86.6%) | 15 (74%) | 16 (38.1%) | 2551 (78%) | 255 (83%) | 38 (100%) |
| Ivabradine | 57 (44.9%) | 25 (83.3%) | 19 (66%) | 41 (48.8%) | 2565 (79%) | 243 (80%) | 39 (93%) |
| ***ACEI/ARB***  Placebo  Ivabradine | 86 (67.7%)  88 (69.3%) | N/A | N/A | N/A | N/A | N/A | N/A |
| ***ARB*** |  |  |  |  |  |  |  |
| Placebo | 20 (15.7%) | N/A | N/A | 14 (33.3%) | 472 (14%) | 36 (12%) | 1 (3%) |
| Ivabradine | 31 (24.4%) | N/A | N/A | 18 (21.4%) | 455 (14%) | 51 (17%) | 3 (7%) |
| ***Diuretic*** |  |  |  |  |  |  |  |
| Placebo | 116 (91.3%) | 29 (96.6%) | N/A | 32 (76.2%) | 2695 (83%) | 266 (87%) | 32 (84%) |
| Ivabradine | 114 (89.8%) | 30 (100%) | N/A | 71 (84.5%) | 2719 (84%) | 264 (87%) | 35 (83%) |
| ***Aldosterone antagonist*** |  |  |  |  |  |  |  |
| Placebo | 91 (17.7%) | N/A | N/A | 22 (52.4%) | 1941 (59%) | 218 (71%) | 14 (37%) |
| Ivabradine | 106 (83.5%) |  |  | 46 (54.8%) | 1981 (61%) | 224 (74%) | 19 (45%) |

Supplement Table 2. Baseline Characteristics of Included Studies

| **Studies** | **Tsutsui et al 2019 ^[22]^** | **Sarullo et al 2010 ^[24]^** | **Mansour et al 2011 ^[21]^** | **Tsutsui H et al 2016 ^[23]^** | **SHIFT 2010 ^[20]^** | **Tardif JC 2011 ^[26]^** | **Volterrani, M et al 2011 ^[27]^** |
| --- | --- | --- | --- | --- | --- | --- | --- |
| ***Study Arm***  Placebo  Ivabradine | 127  127 | 30  30 | 23  30 | 42  84 | 3290  3268 | 304  307 | 38  42 |
| ***Age***  Placebo  Ivabradine | 60.1±13.7  61.2±13.3 | 52.9±4.9  52.1±6.1 | 52±13  47±13 | 59.4±12.7  58.8±13.5 | 60.1±11.5  60.7±11.2 | 59.1±11.1  60.4±10.9 | 66.7±10.1  66.5±9.2 |
| ***Male/Females (%)***  Placebo  Ivabradine | 80.3/19.7  84.3/15.7 | 74/26  74/24 | 61/39  60/40 | 81/19  88.1/11.9 | 77/23  76/24 | 82/18  80/20 | 68/32  68/32 |
| ***Cardiac Parameters*** |  |  |  |  |  |  |  |
| Ejection Fraction (%)  Placebo  Ivabradine | 26.6±6.1  27.9±5.3 | 29.9 ± 6  30.6 ± 6 | 32.3 ±6.2  30.2 ± 5.6 | 28.5 ±4.9  28.5± 5.2 | N/A | 31.6±9.3  32.3±9.1 | 26±5.0  28±4.8 |
| Heart Rate (beats/min)  Placebo  Ivabradine | 82.7±8.1  82.1±7.2 | 75± 3  76± 5 | 84±10  96± 15 | 81.5± 7.4  83.1 ± 7.3 | 80.1± 9.8  79.7± 9.5 | 78.8±9.2  78.4±9.0 | 76.7±12.8  75.7±12.5 |
| ***Echocardiographic Parameters*** |  |  |  |  |  |  |  |
| LVESD (mm)  Placebo  Ivabradine | N/A | 54±5  55±6 | 58.3±6.5  57.1±8.3 | N/A | N/A | N/A | N/A |
| LVEDD (mm)  Placebo  Ivabradine | N/A | 66.4±4  67.7±3 | 68.1±5.7  67.0±8.4 | N/A | N/A | N/A | N/A |
| LVEDV (ml)  Placebo  Ivabradine | N/A | 198±13  197±14 | 188  186 | N/A | N/A | 174.7±67.6  178.4±63.4 | N/A |
| LVESV (ml)  Placebo  Ivabradine | N/A | 134±13  133±15 | 58.3±6.5  57.1±8.3 | N/A | N/A | 122±59.8  123.8±55.6 | N/A |
| ***Exercise Capacity*** |  |  |  |  |  |  |  |
| Exercise Duration (min)  Placebo  Ivabradine | N/A | 15.1 ±2.2  14.8± 2.5 | 3.8 ± 2.7  5.5± 4.4 | N/A | N/A | N/A | N/A |
| ***Quality of Life (QoL)*** |  |  |  |  |  |  |  |
| Minnesota Questionnaire Score (MLWFH)  Placebo  Ivabradine | N/A | 30.6±2.1  30.9±2.3 | 60.3±5.5  58.8±7.2 | N/A | N/A | N/A | N/A |

Supplement Table 3. Post-Follow up Outcomes

| **Studies** | **Tsutsui H et al 2019 ^[22]^** | **Sarullo et al 2010 ^[24]^** | **Mansour et al 2011 ^[21]^** | **Tsutsui H et al 2016 ^[23]^** | **SHIFT 2010 ^[20]^** | **Tardif JC 2011 ^[26]^** | **Volterrani, M et al 2011 ^[27]^** |
| --- | --- | --- | --- | --- | --- | --- | --- |
| ***Study Arm***  Placebo  Ivabradine | 127  127 | 30  30 | 23  30 | 41  81 | 3264  3241 | 199  204 | 38  42 |
| ***Ejection Fraction (%)***  Placebo  Ivabradine | 33.3±13  38.9±12.8 | 30.1±7  35.4±5 | 34.1±6.7  36.4±8.3 | 31 ±8.8  34.4± 9.5 | N/A | 31.5±10.0  34.7±10.2 | No significant difference between 2 groups |
| ***Heart Rate (bpm)***  Placebo  Ivabradine  ***Echocardiographic Parameters*** | 76.6±10.7  66.7±11.4 | 74± 5  63± 3 | 81±7.7  72± 13 | 79.8±9.4  66.6±7.25 | 74± 8.1  65±10.9 | N/A | 64.3±5.4  58.1±5.4 |
| LVEDD (mm)  Placebo  Ivabradine | N/A | 67.5±3  66.9±2 | 67±1  65±1 | N/A | N/A | N/A | N/A |
| LVEDV (ml)  Placebo  Ivabradine  LVESD (mm)  Placebo  Ivabradine  LVESV (ml)  Placebo  Ivabradine | N/A  N/A  N/A | 199±12  194±16  55±4  52±3  136±12  124±14 | 203±1  173±1  56.8±1  53±1  137±1  110±1 | N/A  N/A  N/A | N/A  N/A  N/A | 171.7±63.8  163.7±60.6  N/A  120.9±56.4  110.8±54.6 | N/A  N/A  N/A |
| ***Exercise Capacity (min)***  Exercise Duration  Placebo  Ivabradine | N/A | 15.4±2.6  28.2± 3.5 | 5 ± 2.9  9.2± 4 | N/A | N/A | N/A | N/A |
| 6 Minutes Walking Test (6MWT)  Placebo  Ivabradine | N/A | N/A | N/A | N/A | N/A | N/A | 435.7±125.3  453.1±87.4 |
| ***Quality of Life*** |  |  |  |  |  |  |  |
| MLWFH  Placebo  Ivabradine | N/A | 31.2±2.6  37.5±1.9 | 51.6±5.2  46.5±3.3 | N/A | N/A | N/A | N/A |
